# Supplementary material for: Molecular Weight Identification of Compounds Involved in the Fungal Synthesis of AgNPs: Effect on Antimicrobial and Photocatalytic Activity
Source: Antibiotics (Basel). 2022 May 5;11(5):622. doi: 10.3390/antibiotics11050622 (PMC9138036; doi:10.3390/antibiotics11050622)
Supplement: Supplementary file 1 [file antibiotics-11-00622-s001.zip › antibiotics-1663810-supplementary.pdf]

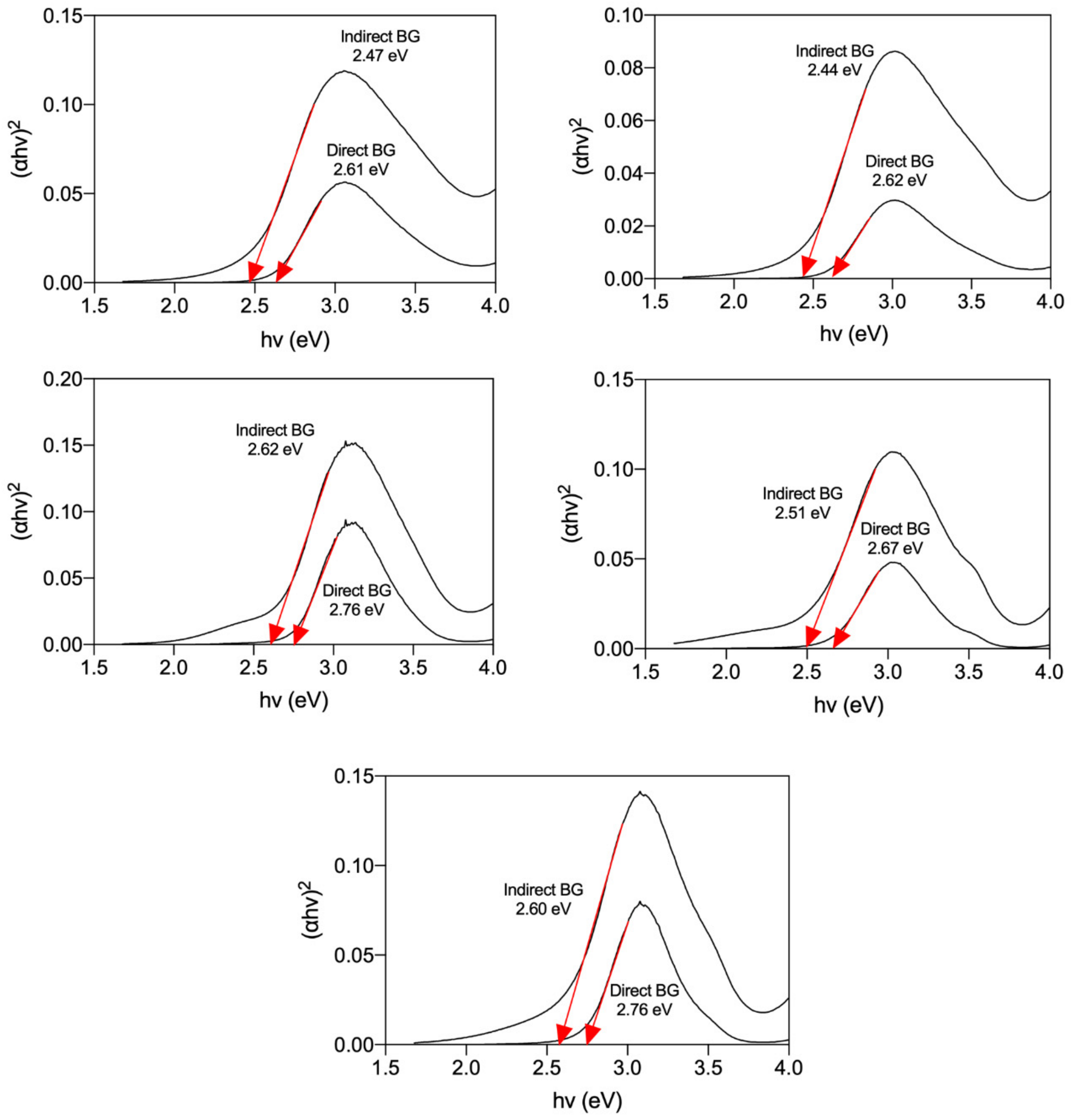

Figure S1. Band gap of silver nanoparticles obtained using different mycelia-free water extract fractions.
